# Changes of bile acids and resting energy expenditure after laparoscopic cholecystectomy in type 2 diabetes patients: a prospective study

**DOI:** 10.1186/s13098-022-00880-3

**Published:** 2022-07-30

**Authors:** Haixin Yin, Weijie Chen, Xiaodong He, Jianping Zeng

**Affiliations:** 1 Hepato-pancreato-biliary Center, School of Clinical Medicine, Beijing Tsinghua Changgung Hospital, Tsinghua University, Beijing, 102218 People’s Republic of China; 2 Department of General Surgery, Chinese Academy of Medical Sciences& Peking Union Medical College, Peking Union Medical College Hospital, Shuaifuyuan 1#, Dongcheng District, Beijing, 100730 People’s Republic of China

**Keywords:** Cholecystectomy, Total bile acids, Type 2 diabetes, Energy, Metabolism

## Abstract

**Background:**

We aimed to investigate changes of bile acids and resting energy expenditure (REE) in patients with type 2 diabetes mellitus (T2DM) after laparoscopic cholecystectomy (LC) and the role in metabolic homeostasis.

**Methods:**

From December 2019 to December 2021, a total of 77 T2DM patients with gallbladder polyps were included in our study. Among them, 40 patients who underwent LC were enrolled into the cholecystectomy group, and 37 patients who did not undergo LC were enrolled into the control group. Preoperative and 6-months postoperative demographic data, body weight, food intake, effects on diabetes control, and biomedical variables were recorded and compared.

**Results:**

The mean level of total bile acids (TBA) was higher than that in the control group (*P* = 0.033) and increased significantly after LC compared to baseline (*P* = 0.029). The REE level in the cholecystectomy group was higher than that in the control group (*P* = 0.032) and increased compared to the baseline (*P* = 0.011). The utilization of carbohydrates increased significantly after LC (P < 0.001) while the utilization of fat decreased (P < 0.001). The mean level of fasting plasma glucose (*P* = 0.004), hemoglobin A1C (*P* < 0.001), and homeostasis model assessment-insulin resistance (*P* = 0.045) decreased after LC. The mean level of total cholesterol (*P* = 0.003) and low-density lipoprotein cholesterol significantly decreased (*P* = 0*.*021), whereas the level of high-density lipoprotein cholesterol increased (*P* < 0.001).

**Conclusions:**

The level of REE and TBA increased after LC in patients with T2DM, and the glucose and lipid metabolism improved.

*Trial registration* This study was registered in the Chinese Clinical Trial Registry on November 30, 2018, registered number: ChiCTR1900027823.

## Introduction

Laparoscopic cholecystectomy (LC) is the commonly performed procedure for the surgical treatment of patients with gallbladder disease [1]. Currently, the gallbladder is not considered just as storage of bile, previous researches demonstrated that gallbladder removal can lead to numerous metabolic changes [2, 3]. Studies reported bile acids (BAs) level was increased after cholecystectomy in mice [4, 5], and circulated faster by continuously secreting into the duodenum [6], thereby increasing basal metabolic rate through G-protein coupled bile acids receptor-dependent mechanisms. While, studies also showed that BAs level remained unchanged after cholecystectomy [6, 7], or decreased [8] may be due to the increased bile loss in feces [9]. Therefore, the changes in BAs after cholecystectomy were still controversial.

Type 2 diabetes mellitus (T2DM) is currently a global health crisis that leads to a decrease in quality of life for patients and a heavy economic burden on society [10, 11]. Over decades, researchers have devoted themselves to exploring the pathogenesis of diabetes, and looking for effective predictors and ways to improve diabetes and its complications. While T2DM is a complex whole-body metabolic abnormality, macroscopically involving age, obesity, diets, and lifestyle, also genetic architecture, signaling pathways, inflammation, endothelial dysfunction, and iron overload in microscopic [12]. To date, there is not a single approach to cure or even remit diabetes, a “cocktail therapy” is still the mainstream treatment. Therefore, a whole and in-depth understanding of pathogenesis and metabolic characteristics in diabetes are conducive to developing effective treatments.

Currently, most studies focus on reducing sugar intake and accelerating blood glucose conversion, but there are few studies on whether increased energy consumption can help reduce blood glucose in diabetics. Since a study in humans showed resting energy expenditure (REE) increased after LC [13]. Increased energy expenditure may decrease the glucose level since blood glucose is the main source of energy in the body. While this human study was a short-term study (within 3 postoperative days), the results were inevitably affected by the postoperative stress response and inflammation. Therefore, the REE in long-term effects remains unclear, and these changes in patients with T2DM have been even less reported. We aimed to investigate the long-term effects of LC on total bile acids (TBA) and REE in patients with T2DM and the role in metabolic homeostasis.

## Patients and methods

### Patients

This study included T2DM patients with asymptomatic gallbladder polyps in the department of general surgery at Peking Union Medical College Hospital (PUMCH, Beijing, China) from December 2019 to December 2021. A total of 104 consecutive outpatients met the inclusion criteria, of whom 77 (74%) agreed to participate in the study. Then patient preference design randomized controlled trial was used to minimize the selection bias [14]. Patients with surgical indications of gallbladder polyps who have a clear preference for surgical treatment were enrolled into the experimental group. The rest of the patients with indications cannot decide whether to perform a surgery or not, they were randomized and enrolled into the experimental group or control group. During the same period, T2DM patients in outpatient service without indications of gallbladder polyps matched for gender, age, and body mass index (BMI) were enrolled into the control group. We estimated that approximately 36 patients in each group would be needed to demonstrate statistically significant changes in the glucose profile. T2DM was diagnosed according to guidelines from the American Diabetes Association: (1) fasting plasma glucose (FPG) ≥ 7 mmol/L (126 mg/dL); or (2) random plasma glucose ≥ 11.1 mmol/l (200 mg/dl); or 3) 2-h plasma glucose ≥ 11.1 mmol/L (200 mg/dL) during an oral glucose tolerating test; or 4) hemoglobin A1C (A1C) ≥ 6.5% (48 mmol/mol) [15].

Cholecystolithiasis patients who underwent LC were excluded because there is an association between gallstone disease and metabolic syndromes [16] and the inflammation involved in cholecystitis might affect glucose metabolism [17]. Other exclusion criteria were as follows: (1) coexisting malignant diseases; (2) gallbladder polyps confirmed as malignant; (3) complications such as fistula, bile duct injury, and infection; (4) debilitating disease, unresolved psychiatric illness, pregnancy, or substance abuse, which might affect metabolism and follow-up work; (5) lipid-lowering agents or changing dietary habits after surgery; (6) patients who dropped out of the study.

This study was approved by the Ethics Committee of PUMCH at the Chinese Academy of Medical Sciences and Peking Union Medical College, and registered in the Chinese Clinical Trial Registry, registered number: ChiCTR1900027823. Informed consents were obtained after a detailed explanation of the study.

### Methods

Preoperative and 6-months postoperative data regarding sex, age, height (to within 0.1 cm), body weight (to within 0.1 kg), BMI, food intake, comorbidities, operation details, and diabetes mellitus, including disease duration and medication use, were recorded and analyzed. The change in food intake amount was assessed using the 2005 Block Food Frequency Questionnaire, which has been used in numerous previous weight-loss intervention trials, including the Diabetes Prevention Program [18].

### Resting energy expenditure (REE)

The REE and energy substrate (carbohydrates and fat) consumption were measured with a Metabolism Testing Equipment (COSMED Quark PFT Ergo, Italy). After fasting for 12 h, the patients were tested at an ambient temperature of 25 degrees centigrade. The patient lay flat on the test bed without any activity, after 5 min of adaptation, the oxygen consumption and carbon dioxide output were obtained from a shield covering the head, thereby the REE was calculated. The whole procedure took approximately 20 min.

### Biomedical parameters

Biomedical parameters obtained included TBA, FPG, A1C, insulin, c-peptide, total cholesterol (TC), triglyceride (TG), high-density lipoprotein cholesterol (HDL-C), and low-density lipoprotein cholesterol (LDL-C) levels. The homeostasis model assessment-insulin resistance (HOMA-IR) was calculated using the formulas: HOMA-IR = FPG (mmol/L) × insulin (mIU/ml) / 22.5, homeostasis model assessment—beta cell function (HOMA-β) was calculated as follow: HOMA-β = 20 × insulin (mIU/ml) / [FPG (mmol/L) – 3.5] [19].

### Definition of remission in T2DM

Enrolled patients with T2DM controlled their glucose with either subcutaneous insulin or oral hypoglycemic agents which included metformin, acarbose, glimepiride, pioglitazone, and gliquidone. The usage of antidiabetic medication was considered a reduction if the amount is reduced compared to that before. At the end of the study patients who had A1C of < 6.0% without any antidiabetic drugs were defined as “in remission”, patients who had A1C of < 6.0% with a reduction of antidiabetic medication were defined as “improved”, patients with an A1C concentration > 6.0% or without a reduction of antidiabetic medication was defined to “unimproved” [19, 20].

### Statistical analysis

We conducted the statistical analysis using SPSS Statistics software (version 24.0, IBM, USA) and drafted histograms using the GraphPad software (version 7.0, GraphPad Prism, USA). Quantitative data are shown as the mean ± standard deviation, and differences in continuous variables before and after surgery were assessed by repeated-measures ANOVA. The multivariate analysis and chi-square test were used to compare each variable between the cholecystectomy and the control group. All statistics were 2-tailed, and *P* values less than 0.05 were considered statistically significant.

## Results

### Patients

From December 2019 to December 2021, a total of 77 T2DM patients were included in this study, 40 of them underwent surgical treatment and enrolled in the cholecystectomy group, and 37 patients without surgical indications were enrolled into the control group. The preoperative clinical characteristics of both groups in age, sex, BMI, duration of diabetes, and other biochemical indicators were compared in Table 1. There were no subjects who dropped out throughout the study.

**Table 1:** Baseline characteristics of the enrolled patients with type 2 diabetes in both groups

Characteristics	Cholecystectomy (n = 40)	Control (n = 37)	*p* value
Age (years)	55.9 ± 14.4	51.2 ± 12.0	0.125
Male/Female (n)	17/23	19/18	0.583
BMI (kg/m^2^)	24.4 ± 3.3	25.2 ± 3.0	0.287
Food intake (kcal)	1925.4 ± 458.2	2015.3 ± 369.1	0.349
Duration of diabetes (month)	20.9 ± 12.9	19.9 ± 13.6	0.726
TBA (μmol/L)	3.3 ± 2.2	3.3 ± 2.9	0.987
REE (kcal)	1292.0 ± 260.4	1353.0 ± 315.6	0.356
FAT (%)	57.0 ± 19.0	54.2 ± 19.8	0.530
CHO (%)	43.5 ± 18.9	46.3 ± 19.8	0.522
FPG (mmol/L)	5.7 ± 1.4	5.6 ± 1.4	0.891
A1C (%, mmol/mol)	6.0 ± 1.2 (42.2 ± 11.1)	6.0 ± 1.3 (41.9 ± 13.2)	0.935
Insulin (uIU/ml)	10.0 ± 4.9	10.3 ± 5.9	0.831
C-peptide (ng/ml)	1.5 ± 0.6	1.6 ± 0.7	0.817
HOMA-IR	2.5 ± 1.6	3.1 ± 2.2	0.179
HOMA-β	124.5 ± 92.8	123.6 ± 58.9	0.960
TC (mmol/L)	4.8 ± 1.0	4.7 ± 0.9	0.732
TG (mmol/L)	1.4 ± 0.7	1.5 ± 0.8	0.656
HDL-C (mmol/L)	1.2 ± 0.3	1.1 ± 0.2	0.080
LDL-C (mmol/L)	3.0 ± 1.0	3.0 ± 0.8	0.827
Anti-diabetic medicine			
Insulin/OHA (n)	17/23	15/22	0.862

Data are shown as the mean ± SD.* indicates *P* < 0.05, ** indicates *P* < 0.01, compared between the two preoperative groups

*BMI* body mass index, *FPG* fasting plasma glucose, *A1C* hemoglobin A1c, *TC* total cholesterol, *TG* triglyceride, *HDL-C* high-density lipoprotein cholesterol, *LDL-C* low-density lipoprotein cholesterol, *TBA* total bile acid, *HOMA-IR* homeostasis model assessment-insulin resistance, *HOMA-β* homeostasis model assessment-beta-cell function, *OHA* oral hypoglycemic agents, *REE* resting energy expenditure, *CHO* carbohydrate

### Operation details

All included patients in the cholecystectomy group were admitted to the hospital and successfully underwent LC without postoperative complications. The postoperative pathological results verified that all specimens were benign polyps, including 33 cases with single polyps and 7 cases with multiple polyps. The duration of the operation was 22.6 ± 10.6 min, intraoperative blood loss was 45.4 ± 17.6 ml, and the duration of hospital stay was 2.6 ± 0.9 days. All patients were followed up for at least 6 months.

### Bodyweight and food intake

There was no significant difference in BMI between groups at the baseline (Table 1) and 6 months after surgery (Table 2), and there were no differences in the before-after analysis of body weight in the cholecystectomy group and control group (Table 2). Food intake was estimated by questionnaires or telephone interviews, and the composition did not change much at 6 months after surgery. The daily food intake amounts between groups did not change at baseline (Table 1) and 6 months after surgery (Table 2), nor were that before and after in the cholecystectomy group and control group (Table 2).

**Table 2:** Comparison of variables before and after for each group, and between groups at 6 months after surgery

Variable	Cholecystectomy (n = 40)	*P*^*#*^	Control (n = 37)	*P*^*&*^	*P*
BMI (kg/m^2^)	24.4 ± 3.3	0.881	25.2 ± 3.1	0.819	0.309
Food intake (kcal)	1862.7 ± 358.2	0.497	1988.7 ± 476.5	0.789	0.192
TBA (µmol/L)	4.3 ± 3.0	0.029*	3.1 ± 1.9	0.654	0.033*
REE (kcal)	1430.0 ± 257.5	0.011*	1291.3 ± 298.6	0.263	0.032*
FAT (%)	39.8 ± 21.1	< 0.001 **	49.9 ± 19.5	0.194	0.032*
CHO (%)	60.5 ± 20.9	< 0.001 **	50.6 ± 19.4	0.201	0.036*
FPG (mmol/L)	4.9 ± 1.0	0.004**	5.3 ± 0.7	0.138	0.047*
A1C (%, mmol/mol)	5.2 ± 0.7 (32.8 ± 7.6)	< 0.001 **	5.6 ± 0.6 (37.3 ± 6.9)	0.064	0.008 **
Insulin (µIU/mL)	8.0 ± 4.3	< 0.001 **	9.8 ± 5.7	0.064	0.130
C-Peptide (ng/mL)	1.5 ± 0.6	0.962	1.5 ± 0.5	0.571	0.715
HOMA-IR	2.0 ± 1.3	0.045*	2.6 ± 1.5	0.177	0.043*
HOMA-β	99.8 ± 77.1	0.059	126.5 ± 74.4	0.827	0.126
TC (mmol/L)	4.3 ± 0.9	0.003**	4.7 ± 0.9	0.864	0.040*
TG (mmol/L)	1.4 ± 0.8	0.924	1.6 ± 0.9	0.681	0.475
HDL-C (mmol/L)	1.8 ± 0.3	< 0.001 **	1.2 ± 0.3	0.026	< 0.001 **
LDL-C (mmol/L)	2.6 ± 0.9	0.028*	2.8 ± 0.8	0.229	0.221

Data are shown as the mean ± SD.* indicates *P* < 0.05; ** indicates *P* < 0.01, *P*^*#*^ compared before and after for cholecystectomy group; *P*^*&*^ compared before and after for control group; *P* compared between the two postoperative groups

*BMI* body mass index, *FPG* fasting plasma glucose, *A1C* hemoglobin A1c, *TC* total cholesterol, *TG* triglyceride, *HDL-C* high-density lipoprotein cholesterol, *LDL-C* low-density lipoprotein cholesterol, *TBA* total bile acid, *HOMA-IR* homeostasis model assessment-insulin resistance, *HOMA-β* homeostasis model assessment-beta-cell function, *OHA* oral hypoglycemic agents, *REE* resting energy expenditure, *CHO* carbohydrate

### Changes of TBA

**Fig. 1:**
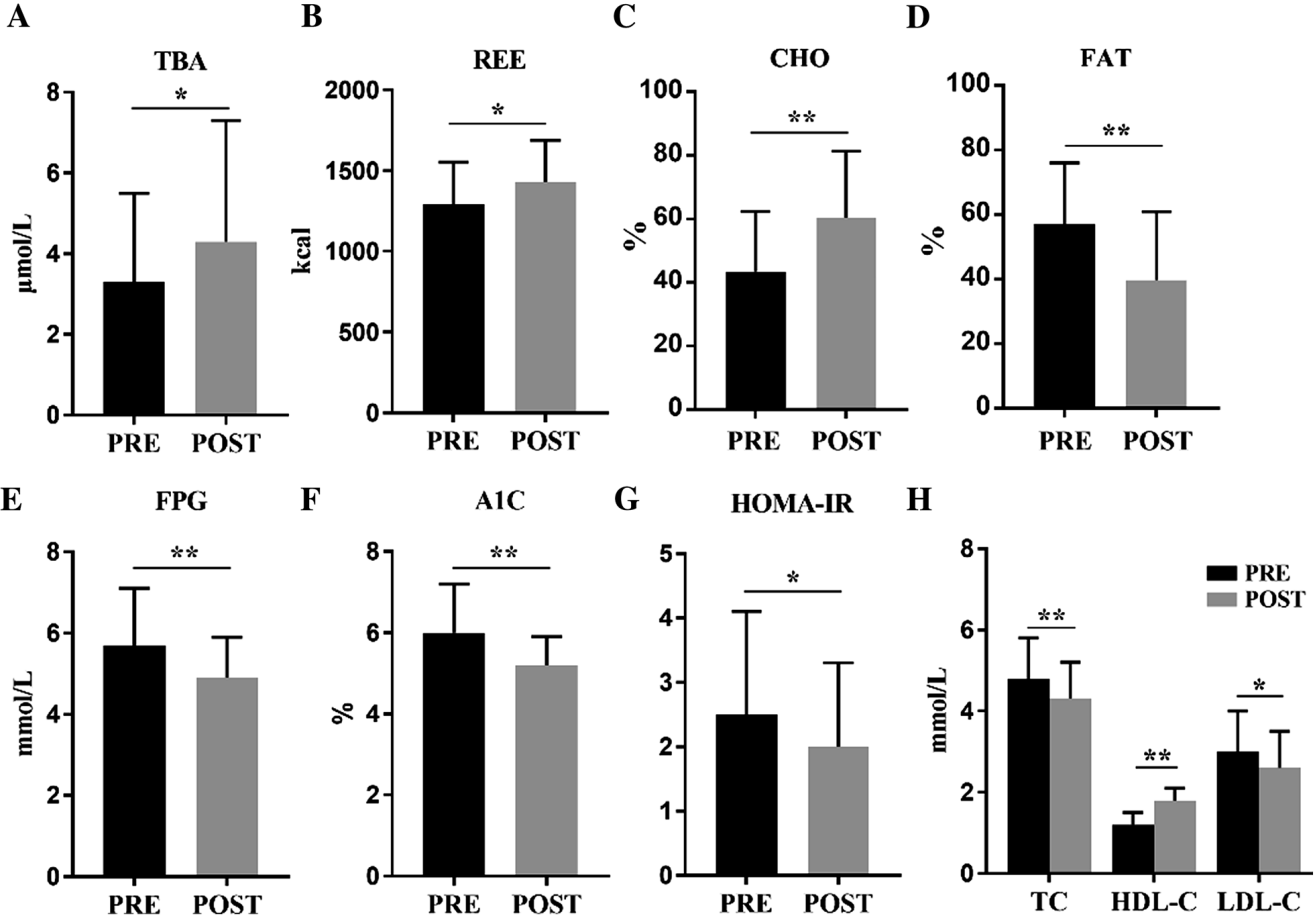
Characteristic changes in the metabolism of energy, glucose, and lipid in cholecystectomy group after LC. The level of TBA (**A**) and REE (**B**) increased after surgery, and consumption of carbohydrates increased (**C**) while fat consumption decreased (**D**). The level of FPG (**E**), A1C (**F**), and HOMA-IR (**G**) decreased 6 months after LC. For lipid metabolism (**H**), TC and LDL-C levels decreased after surgery, and HDL-C level increased. PRE, preoperative; POST, postoperative; TBA, total bile acids; REE, resting energy expenditure; CHO, carbohydrate; FPG, fasting plasma glucose; A1C, hemoglobin A1c; HOMA-IR, homeostasis model assessment-insulin resistance; TC, total cholesterol; HDL-C, high-density lipoprotein cholesterol; LDL-C, low-density lipoprotein cholesterol. Data are shown as the mean ± SD, * indicates P < 0.05, ** indicates P < 0.01, compared between preoperative and postoperative

The mean level of TBA in the cholecystectomy group was higher than that in the control group (*P *= 0.033) (Table 2), and it was increased significantly 6 months after LC compared to the baseline (*P *= 0.029) (Fig. 1A). TBA level in the control group did not change in the before-after analysis (*P *= 0.654) (Table 2).

### Effect on energy metabolism

The REE level in the cholecystectomy group was higher than that in the control group at 6 months after LC (*P *= 0.032) and increased significantly compared to the baseline (*P *= 0.011) (Fig 1B). Carbohydrates and fat, the main sources of body energy [21], showed opposite changes. The utilization of carbohydrates was higher in the cholecystectomy group (*P *= 0.036) (Table 2) and increased significantly compared to baseline (P < 0.001) (Fig 1C) while the utilization of fat in the cholecystectomy group was lower than that in the control group (*P *= 0.032) (Table 2) and decreased compared to baseline (P < 0.001) (Fig 1D). The REE level (*P *= 0.263) and utilization of carbohydrate (*P *= 0.201) and fat (*P *= 0.194) in the control group did not change compared to its baseline (Table 2).

### Effect on glucose metabolism

Glucose metabolism in T2DM patients improved after LC. According to the definitions, 5 patients (12.5%) were “in remission”, 13 (32.5%) were at an “improved” status 6 months after surgery. While the dosage of antidiabetic drugs used in the control group did not change.

The biomedical indicators compared between groups after surgery were showing in Table 2. The mean level of FPG was lower than that in the control group 6 months after surgery (*P* = 0.047) and decreased compared to the baseline (*P* = 0*.*004) (Fig 1e). The A1C level was also lower compared to the baseline (*P* < 0.001) (Fig 1F) and control group (*P* = 0.008). Additionally, the average value of HOMA-IR was lower than that in the control group (*P* = 0.043) and baseline (*P* = 0.045) (Fig 1G). There was no significant difference in the HOMA-β value compared to the baseline (*P* = 0.059) and control group (*P* = 0.126). All the glucose metabolic variables in the control group did not change in comparison with their baseline values (Table 2).

### Effect on lipid metabolism

The metabolic changes of lipid in the cholecystectomy group after LC were shown in Fig 1H. The mean level of TC (*P* = 0.002) and LDL-C (*P* = 0*.*028) were significantly decreased compared to their baseline at 6 months after surgery. While the average level of HDL-C increased significantly compared to the baseline (*P* < 0.001). Compared to the control group, the TC level in the cholecystectomy group was lower (*P* = 0.040) (Table 2), while the HDL-C level was higher (*P* < 0.001). There was no significant difference in the TG level compared to the baseline (*P* = 0*.*957) and control group (*P* = 0.475). All the lipid metabolic variables of the control group in comparison with its baseline values were shown in Table 2.

## Discussion

The changes in TBA and REE after cholecystectomy in patients with T2DM have been less reported. Our study showed that the level of TBA and REE increased significantly after LC in patients with T2DM, and the utilization of carbohydrates elevated while that of fat decreased.

BAs played an important role in the elimination of cholesterol and the absorption of vitamins and fats [22]. It was synthesized from cholesterol in the liver, accounting for catabolism of approximately 50% of the daily cholesterol output. BAs are stored in the gallbladder and secreted into the intestine when a meal is ingested, yet 95% of BAs are reabsorbed and transported back to the liver via the portal vein, escaped BAs were converted to secondary BAs by intestinal microbiota and excreted in the feces. This system is known as enterohepatic circulation [23]. The rhythmic filling and emptying of the gallbladder control the flow of bile into the intestine and thereby the enterohepatic circulation. Normally, the pool and circulation of BAs maintain a dynamic balance, while it would be disrupted by biliary intervention or in pathological conditions.

The gallbladder was considered merely to concentrate and store bile by absorbing water and ions previously, while recent studies showed that cholecystectomy would affect the metabolism of BAs. Several studies have demonstrated that TBA levels increase markedly after cholecystectomy [5, 24], while other studies showed BAs remained unchanged [6] or decreased [8]. In our study TBA level increased significantly after LC, but the underlying mechanism was still not elucidated. Removal of the gallbladder leads BAs to continuously secret into the duodenum, theoretically faster circulated BAs would inhibit the cholesterol 7α-hydroxylase in the liver, the rate-limiting enzyme for bile formation, thereby reducing the bile synthesis. However, increased TBA levels were observed in most studies or at least unchanged. Increased bile loss in feces due to the enhanced enterohepatic circling after cholecystectomy has been demonstrated [9], thereby the bile synthesis compensatory increased. We suspected massive loss of bile led to an excessive bile synthesis, it may play a predominant role in the bile synthesis, but further studies still needed to be performed.

In recent years, increasing attention has been paid to the role of BAs as a signaling molecular, that regulates various hormones and receptors and modulates whole-body metabolic homeostasis [25]. The most widely studied receptors are the farnesoid X receptor (FXR) and the membrane G protein-coupled BA receptors (GPBAR1/TGR5) [26]. FXR stimulates the secretion of fibroblast growth factor 19 into the portal circulation and activates its fibroblast growth factor 4 liver receptor, leading to decreased gluconeogenesis glycemia and improved insulin sensitivity and glucose and lipid metabolism in diabetes [27, 28]. BAs activate TGR5, which is expressed in enteroendocrine L cells and stimulates the secretion of glucagon-like peptide-1, thereby improving liver and pancreatic function, stimulating insulin secretion from β-cells, increasing insulin sensitivity and glucose tolerance [29].

Moreover, BAs are also involved in energy expenditure, in line with previous studies, we found REE increased after cholecystectomy [4, 13]. The activation of TGR5 by BAs induces thyroid hormone deiodinase type 2, which converts the inactive thyroid hormone thyroxine to active triiodothyronine thereby increasing energy expenditure [30]. Moreover, plasm BAs can directly promote heat production in brown adipose tissue and skeletal muscle, which were two important organs for thermogenesis [31, 32]. Energy expenditure is also influenced by an interplay of BAs and intestinal microbiota. The level and composition of TBA affect the gut microbial community abundance and composition [33]. The gut microbiota can digest complex food components and produce signaling molecules, including short-chain fatty acids, lipopolysaccharides, peptidoglycan, etc. Such signaling molecules promoted energy intake, use, and expenditure [34].

Although studies reported glucose deterioration in normal patients after LC [3, 35], this may not be applied to the patients with T2DM due to the damaged metabolic regulation. Our study showed that glucose improved in patients with T2DM after LC, the level of FPG and A1C decreased compared to baseline. While there were no significant changes in food intake and body weight. Therefore, these improvements were unrelated to food intake and weight loss. Increased BAs may be responsible for glucose improvement, according to the abovementioned mechanism, BAs can directly or indirectly activate several gut hormones, thereby improving insulin resistance which was also confirmed by the estimation of HOMA-IR in the present study. Moreover, the proportion of carbohydrates in the REE increased after LC while that of fat decreased. Carbohydrates are the most important source of energy, and approximately 50–70% of the energy used in the body comes from the breakdown of blood glucose. Therefore, a decrease in glucose level is also related to the increased REE level.

With the improvement in glucose metabolism, dyslipidemia was also alleviated, HDL-C levels were elevated after surgery, while TC and LDL-C concentrations were significantly decreased. Dyslipidemia is an important component of diabetes and has received much attention in recent years, but the underlying pathophysiology is complex and still not well understood [36]. Insulin resistance is believed to be the main trigger for diabetic dyslipidemia. Insulin is involved in the synthesis and secretion of lipoprotein, suppresses lipolysis in adipose tissues, regulates the amount of circulating free fatty acids, and inhibits the transcription of microsomal triglyceride transfer protein in the liver, which mediates the transfer of triglycerides to nascent apolipoprotein B, the predominant surface protein of very-low-density lipoprotein. A reduction in insulin resistance positively regulates this process, helping lower the lipoprotein. Therefore, the reduction in insulin resistance may lead to the improvement of dyslipidemia which was confirmed in our study. Additionally, beneficial changes in lipids metabolism were more likely to occur in the dyslipidemic patients after biliary interventions, this had also been proved in several previous studies [9, 37], but these effects were not found in patients with normal lipids metabolism. Nevertheless, the underlying mechanism of this difference between dyslipidemic and non-dyslipidemic patients was still unclear.

There are a few limitations of this study. A randomized study needed to be performed to confirm our results, and the effects on gut hormones also need to be investigated. Nevertheless, the results of our study show that the level of TBA and REE increased after cholecystectomy in patients with T2DM, and indicate the possible role of the BAs and REE in remission of T2DM which may be a potential possibility for the treatment of metabolic diseases. Moreover, considering the improvement of glucose after cholecystectomy in patients with T2DM, we may adopt a positive surgical attitude to these T2DM patients with gallbladder disease in the clinic, either to avoid the higher risk of emergency surgery or maximally control the progress of T2DM and its complications.

## Conclusions

The level of REE and TBA increased after LC in patients with T2DM, the glucose and lipid metabolism improved.

## Data Availability

The datasets generated and/or analyzed during the current study are not publicly available due to personal data protection legislation but are available from the corresponding author on reasonable request.

## Abbreviations

LC: Laparoscopic cholecystectomy
BAs: Bile acids
T2DM: Type 2 diabetes mellitus
REE: Resting energy expenditure
TBA: Total bile acids
BMI: Body mass index
FPG: Fasting plasma glucose
A1C: Hemoglobin A1C
TC: Total cholesterol
TG: Triglyceride
HDL-C: High-density lipoprotein cholesterol
LDL-C: Low-density lipoprotein cholesterol
HOMA-IR: Homeostasis model assessment-insulin resistance
HOMA-β: Homeostasis model assessment – beta-cell function
FXR: Farnesoid X receptor
GPBAR1/TGR5: Membrane G protein-coupled BA receptors
